# Implementation of Electronic Clinical Decision Support for Radiology Referrals: The Role of Governance, Clinician Engagement and Education

**DOI:** 10.1111/1742-6723.70305

**Published:** 2026-06-28

**Authors:** Bruno Di Muzio, Saman Salaran, Ben Morgan, Andrew Woo, Christopher Perry, Alex Jarema, Philippa Hawkings, Peter Cameron, Meng Law

**Affiliations:** ^1^ Department of Radiology The Alfred Melbourne Australia; ^2^ Department of Surgery School of Clinical Sciences at Monash Health, Monash University Melbourne Australia; ^3^ Medical Services, the Alfred Melbourne Australia; ^4^ Emergency and Trauma Centre The Alfred Melbourne Australia; ^5^ Department of Epidemiology and Preventive Medicine School of Public Health and Preventive Medicine, Monash University Melbourne Australia; ^6^ Department of Neuroscience School of Translational Medicine, Monash University Melbourne Australia

**Keywords:** clinical decision support systems, diagnostic imaging utilisation, emergency department, imaging appropriateness, implementation

## Abstract

**Objectives:**

Medical imaging utilisation continues to increase globally, raising concerns regarding sustainability, workforce capacity, patient safety and environmental impact. Electronic Clinical Decision Support (eCDS) systems have been proposed as a strategy to improve imaging appropriateness; however, international experience suggests that technology alone is insufficient for successful adoption. This study describes an organisational implementation strategy for an eCDS programme for imaging referrals and examines early changes in imaging utilisation associated with clinician engagement and education strategies preceding system go‐live.

**Methods:**

An eCDS system was implemented within the electronic medical record at a quaternary referral centre in Melbourne, Australia. The initiative included governance structures, clinician engagement and targeted education to support integration into clinical workflows. Imaging utilisation data, including computed tomography (CT) examinations per emergency department (ED) presentation, were obtained from hospital activity records and analysed descriptively across engagement and deployment phases.

**Results:**

Prior to eCDS go‐live, CT utilisation increased from 32 to 44 examinations per 100 ED presentations. During the engagement phase preceding system deployment, CT ordering declined by 9.1%, coinciding with clinician engagement and education activities. Following eCDS deployment, imaging utilisation trends stabilised.

**Conclusions:**

Successful implementation of eCDS requires more than technological deployment. Governance, clinician engagement and sustained education appear important for supporting adoption of decision support systems and may facilitate early cultural change in imaging utilisation.

## 1 Introduction

Medical imaging is indispensable in modern healthcare, yet its overutilisation remains a global challenge, driving unnecessary costs, exposing patients to avoidable risks and straining radiology services [1, 2, 3]. Electronic Clinical Decision Support (eCDS) systems have emerged as a key strategy to address this issue by embedding evidence‐based guidelines into referral workflows, promoting appropriate imaging and reducing low‐value studies [4, 5, 6].

### 1.1 Challenges in Imaging Selection and eCDS Adoption

Imaging referral patterns are shaped by a complex interplay of factors beyond clinical guidelines, including clinician experience, service resource availability, workflow constraints and patient expectations [7, 8]. In many cases, patients perceive imaging as essential to good care, and clinicians may feel pressured to over‐service or practise defensively to mitigate medico‐legal risk. These behaviours, combined with conflicting incentives between public and private practise, hinder the adoption of evidence‐based imaging practises and may impact the effectiveness of eCDS systems [7, 9].

Several practical barriers compound this challenge:

- Knowledge gaps regarding current imaging guidelines can lead to low‐value or inappropriate tests with limited diagnostic yield [4]. This is often compounded by the increasing complexity of imaging pathways, frequent guideline updates and variability in local access to subspecialty expertise [10].
- Diagnostic uncertainty and complex clinical presentations may lead to imaging requests outside guideline recommendations [9].
- Increasing workloads, funding constraints and the 24/7 nature of emergency work have reduced opportunities for real‐time consultation with radiologists, limiting access to specialist input on imaging appropriateness and protocol selection. As a result, clinicians may rely more heavily on default imaging pathways, reducing tailoring to complex clinical scenarios and increasing the risk of low‐value imaging [8, 11].
- Service constraints, such as restricted access to MRI or ultrasound after hours, often result in alternative tests with higher radiation exposure and lower diagnostic value [1, 10].
- Finally, patient expectations and a growing ‘zero risk’ culture in medicine reinforce the mindset that ‘more is better’, driving defensive imaging and unsustainable utilisation [1, 9, 11].

These challenges underscore the critical role of eCDS in bridging knowledge gaps, supporting evidence‐based decisions and providing an objective framework for discussions with patients. To be effective, the tool must be tailored to local service realities, with recommendations that explicitly account for factors such as modality availability, workforce capacity and workflow constraints [12, 13]. However, implementation‐level barriers, including limited integration with existing clinical systems (i.e., electronic medical records—EMR), suboptimal user interface design, alert fatigue from frequent or low‐value prompts and inadequate education or training, may impair usability, diminish perceived clinical utility and ultimately constrain adoption and impact [14].

### 1.2 Lessons From International Experience

International experience, particularly from large‐scale implementations in the United States and United Kingdom, shows that passive deployment of eCDS rarely succeeds [15, 16, 17]. Without active governance, clinician engagement and structured education, adoption is inconsistent, impact is limited, and in some cases, initiatives fail entirely. These lessons underscore that success depends on organisational leadership, cultural change and sustained stakeholder involvement.

## 2 Methods

### 2.1 Study Design

This descriptive implementation study reports the introduction of an eCDS system for imaging referrals at Alfred Hospital, a quaternary referral centre in Melbourne, Australia, providing comprehensive emergency, inpatient and outpatient radiology services. The study describes the governance structures, stakeholder engagement strategies and organisational processes used to support integration of eCDS into clinical workflows.

Ethics approval for this study was granted by the Alfred Health Human Research Ethics Committee (Project No. 163/25). The study used de‐identified institutional data derived from routine clinical practise, and individual patient consent was waived.

This study was reported in accordance with the Standards for Quality Improvement Reporting Excellence (SQUIRE 2.0) guidelines.

### 2.2 Setting

The hospital has a high‐volume ED and functions as the primary referral centre within a multi‐campus health service. This report primarily reflects the experience of the main referral campus where programme design, deployment and evaluation were undertaken.

### 2.3 Intervention

**FIGURE 1:**
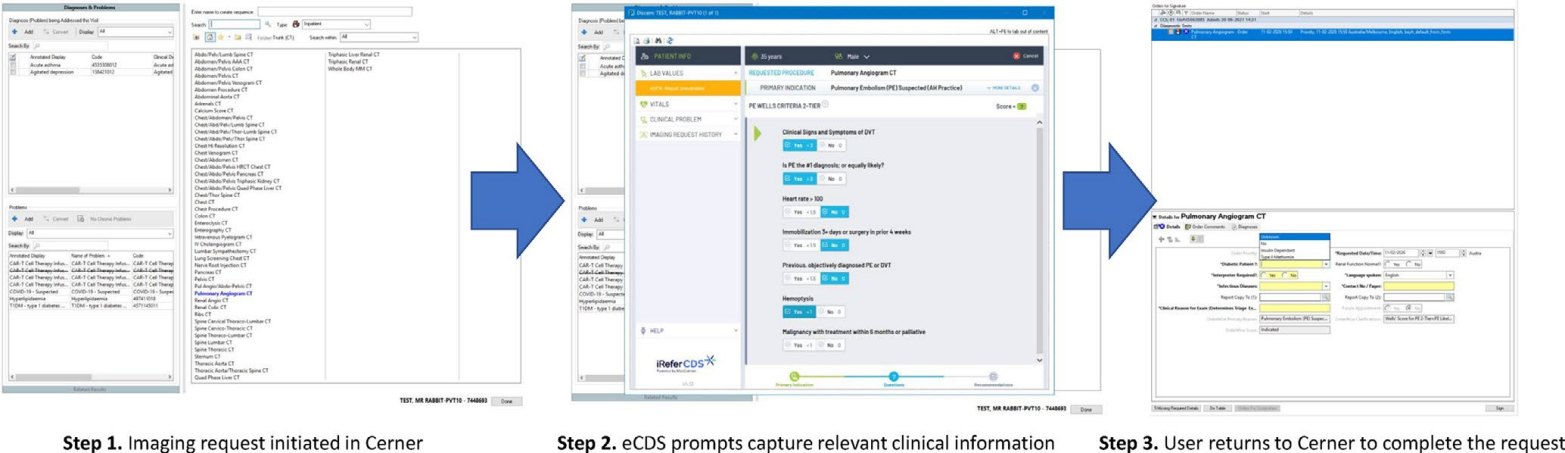
Integration of the eCDS module within the Cerner imaging order workflow. The system prompts for relevant clinical information during order entry before returning the user to the electronic medical record to complete the imaging request.

The intervention involved deployment of an organisation‐led, clinician‐driven eCDS programme aimed at reducing low‐value imaging and improving imaging appropriateness. MedCurrent OrderWise was integrated within the EMR (Cerner Millennium) to provide guideline‐based decision support during imaging order entry. The eCDS module automatically launched during imaging order entry to capture clinical information required for guideline‐based recommendations (Figure 1).

Preparatory engagement activities commenced in September 2023 during the pre‐go‐live engagement phase, followed by a staged eCDS deployment beginning in March 2024. Initial deployment focused on CT requests in the ED due to the observed rise in CT utilisation, with subsequent expansion to inpatient and outpatient settings through a phased approach.

### 2.4 Data Sources and Analysis

Imaging utilisation data were obtained from institutional radiology information systems and hospital activity records. Indicators evaluated included CT examinations per 100 ED presentations and selected diagnostic yield metrics for specific imaging pathways. These indicators were used to illustrate trends before and during the eCDS rollout.

Data were analysed across three periods: pre‐COVID baseline (January 2019–December 2019), pre‐go‐live engagement phase (January 2023–February 2024) and early deployment phase (March 2024–June 2025). Data were analysed descriptively to assess trends in imaging utilisation and referral behaviour across these phases. Organisational processes and implementation strategies were documented to provide a structured account of institutional eCDS adoption.

Quarterly institutional imaging utilisation data from Q1 2017 to Q2 2025 were additionally plotted to provide broader longitudinal context for imaging trends before COVID‐19, during the pandemic period, and throughout the engagement and deployment phases of the eCDS initiative.

## 3 Results

### 3.1 Drivers for Implementation: Rising Imaging Utilisation

The hospital provides comprehensive radiology services, including emergency, inpatient and outpatient imaging, with a high‐volume ED that has experienced significant growth in imaging demand. Internal data indicated that CT examinations per 100 ED presentations increased by 37.5% from pre‐COVID to the pre‐eCDS period, highlighting the need for targeted intervention.

At the time of the pre‐go‐live engagement phase, the hospital faced significant post‐COVID workforce challenges, including radiology staff shortages and low retention rates, particularly amongst radiographers. These issues were compounded by high demand for imaging studies and the operational strain of after‐hours shifts, creating pressure on service delivery and increasing the risk of burnout. Introducing eCDS was therefore not only a strategy to improve appropriateness of imaging but also a critical measure to optimise resource utilisation and support staff wellbeing.

### 3.2 eCDS Deployment and Rollout Strategy

Preparatory clinician engagement and education activities commenced in September 2023 during the pre‐go‐live engagement phase, followed by staged eCDS deployment beginning in March 2024. This phased approach allowed for controlled adoption and iterative refinements across different clinical departments. Initial deployment focused on CT requests in the ED, given the observed increase in CT utilisation before progressive expansion to inpatient and outpatient settings.

During the pre‐go‐live engagement phase, activities focused on clinician education, stakeholder engagement and workflow familiarisation prior to activation of the eCDS platform within the EMR. These activities included departmental presentations, multidisciplinary meetings, targeted discussions with high‐volume referrers and ongoing communication with clinical leadership groups. Clinical champions and super‐users were identified within key departments to support local engagement, provide feedback regarding usability and facilitate integration into clinical workflows.

**FIGURE 2:**
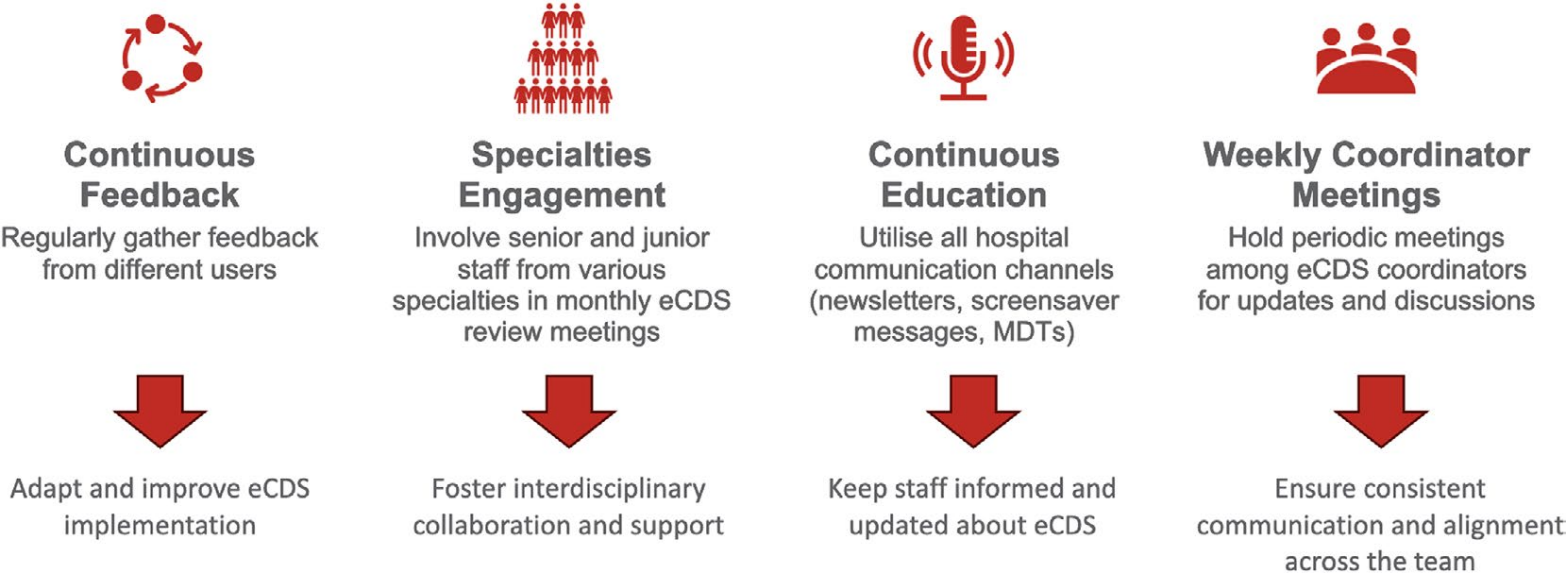
Sustained change management strategies supporting eCDS adoption at Alfred Health. These included continuous feedback from users to refine the system, active engagement of specialties through regular review meetings, ongoing education via multiple communication channels and periodic coordinator meetings (initially weekly, then spaced to fortnightly, monthly and now quarterly) to ensure alignment and consistent communication across the team.

Regular multidisciplinary meetings involving radiology, information technology, operational teams and clinical champions were conducted to refine order pathways, align guideline recommendations with local service availability and address anticipated workflow challenges before staged deployment. Key organisational engagement and change management strategies adopted during this phase are summarised in Figure 2.

**FIGURE 3:**
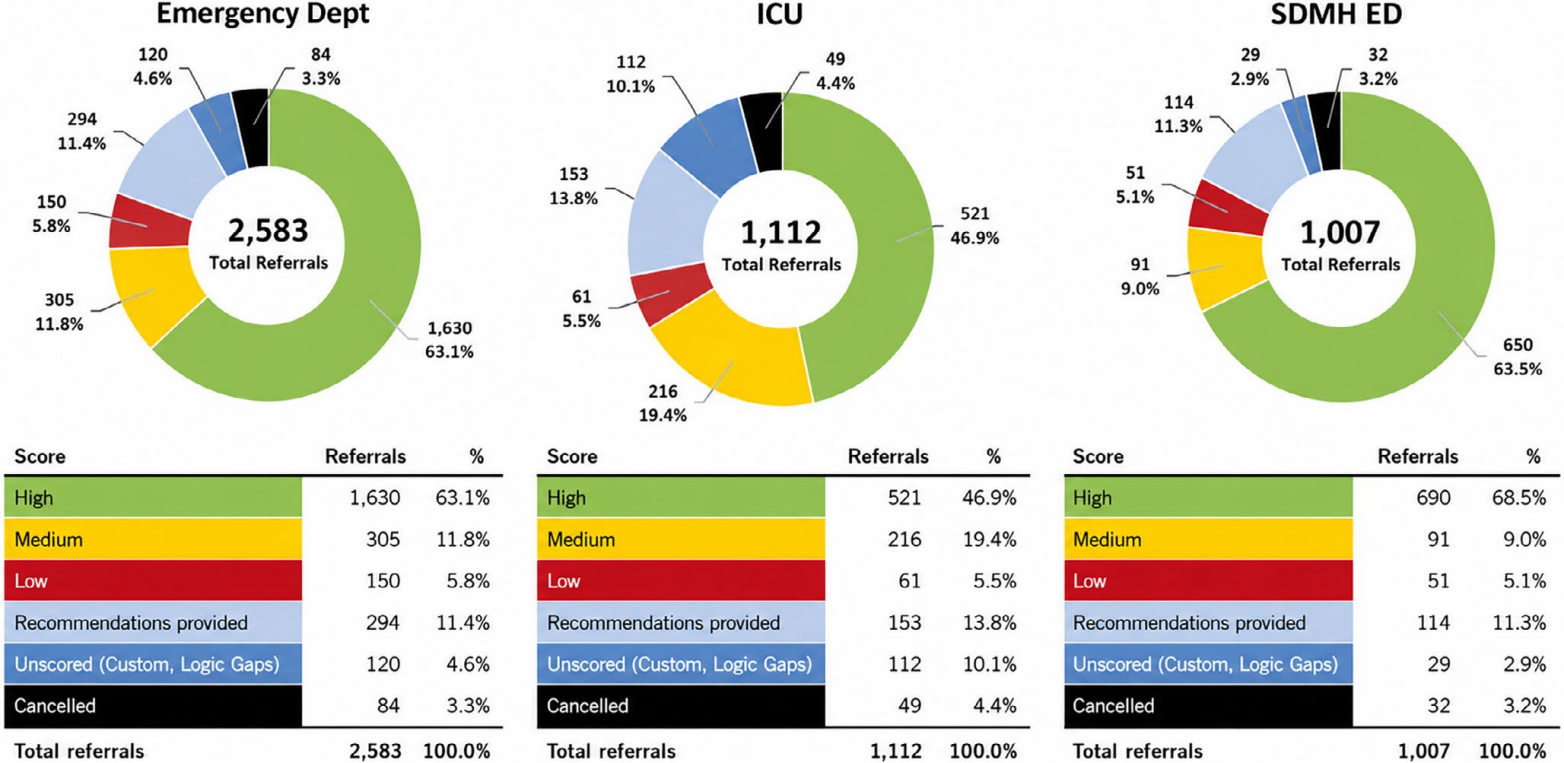
Illustrative example of the eCDS analytics dashboard used for operational monitoring of referral appropriateness across high‐volume requesting departments, including the Emergency Department, Intensive Care Unit (ICU) and Sandringham Hospital Emergency Department (SDMH ED). Circular charts display referral volumes and distributions across appropriateness score categories (high, medium and low), together with recommendation outcomes, unscored referrals and cancellations. Accompanying tables summarise referral counts and proportional distributions across categories. Dashboard data are included to demonstrate operational monitoring capabilities of the eCDS platform and were not analysed as formal study outcomes.

### 3.3 Strategies for Effective eCDS Implementation

Recognising the limitations of passive eCDS deployment reported internationally, we adopted a multifaceted implementation strategy aimed at promoting clinician engagement and sustainable adoption. Key components of this approach are summarised in Table 1.

**TABLE 1:** Organisational strategies supporting adoption of the electronic clinical decision support (eCDS) system.

Strategy	Key elements	Purpose
Education and promotion	Hospital‐wide education campaign including grand rounds, departmental meetings and MDT forums; direct communication with referrers and frontline staff.	Increase awareness of imaging appropriateness and familiarise clinicians with eCDS functionality and workflow integration [7, 12, 18, 19].
Phased rollout and clinical champions	Staged rollout beginning in ICU, followed by ED and other units; identification of clinical champions and super‐users within key specialties to support adoption and workflow integration.	Allow iterative refinement of workflows and leverage peer influence to encourage clinician engagement and adoption [19, 20].
Sustained organisational engagement	Regular feedback loops with clinical specialties, multidisciplinary coordination meetings involving radiology, IT, and clinical champions and ongoing collaboration with the eCDS vendor to refine guidelines and workflows. Education and updates were reinforced through internal communications and departmental engagement (see Figure 2).	Maintain alignment with clinical practise, support continuous system optimisation and sustain clinician engagement beyond the initial rollout [21, 22].
Radiation safety integration	Review of imaging protocols to align with national radiation safety standards; system prompts recommending lower‐radiation alternatives when appropriate.	Promote safe and judicious imaging use whilst maintaining clinical practicality.
Analytics and performance monitoring	Real‐time dashboards monitoring referral behaviour and CDS utilisation (see Figure 3); specialty‐specific feedback reports; localisation of appropriateness pathways to reflect institutional workflows and resource availability.	Identify deviations, guide targeted feedback, support continuous improvement [12, 23, 24].
Governance and policy integration	Integration of eCDS recommendations into institutional imaging protocols, and quality governance processes.	Embed eCDS into routine clinical practise and ensure long‐term sustainability [25].
Recognition and clinician engagement	Internal communication highlighting best practise and departmental successes.	Reinforce clinician engagement and encourage sustained adoption.

Beyond local initiatives, the health network participated in the Safer Care Victoria Sustainable Quality Use of DiagnosticS (SQuDS) programme, which aims to reduce low‐value pathology and imaging whilst improving environmental sustainability. At Alfred Hospital, eCDS was applied to CT pulmonary angiogram (CTPA) requests. This was associated with reduced CTPA utilisation and an increase in CTPA positivity from 11.6% to 12.9%. In 28.8% of cases, eCDS recommended alternative investigations such as chest radiography, lower limb ultrasound or D‐dimer testing based on low pre‐test probability. Notably, this initiative was led by the Emergency Department rather than Radiology, highlighting the value of cross‐disciplinary leadership in promoting appropriate imaging.

### 3.4 Imaging Trends Before and After eCDS

**FIGURE 4:**
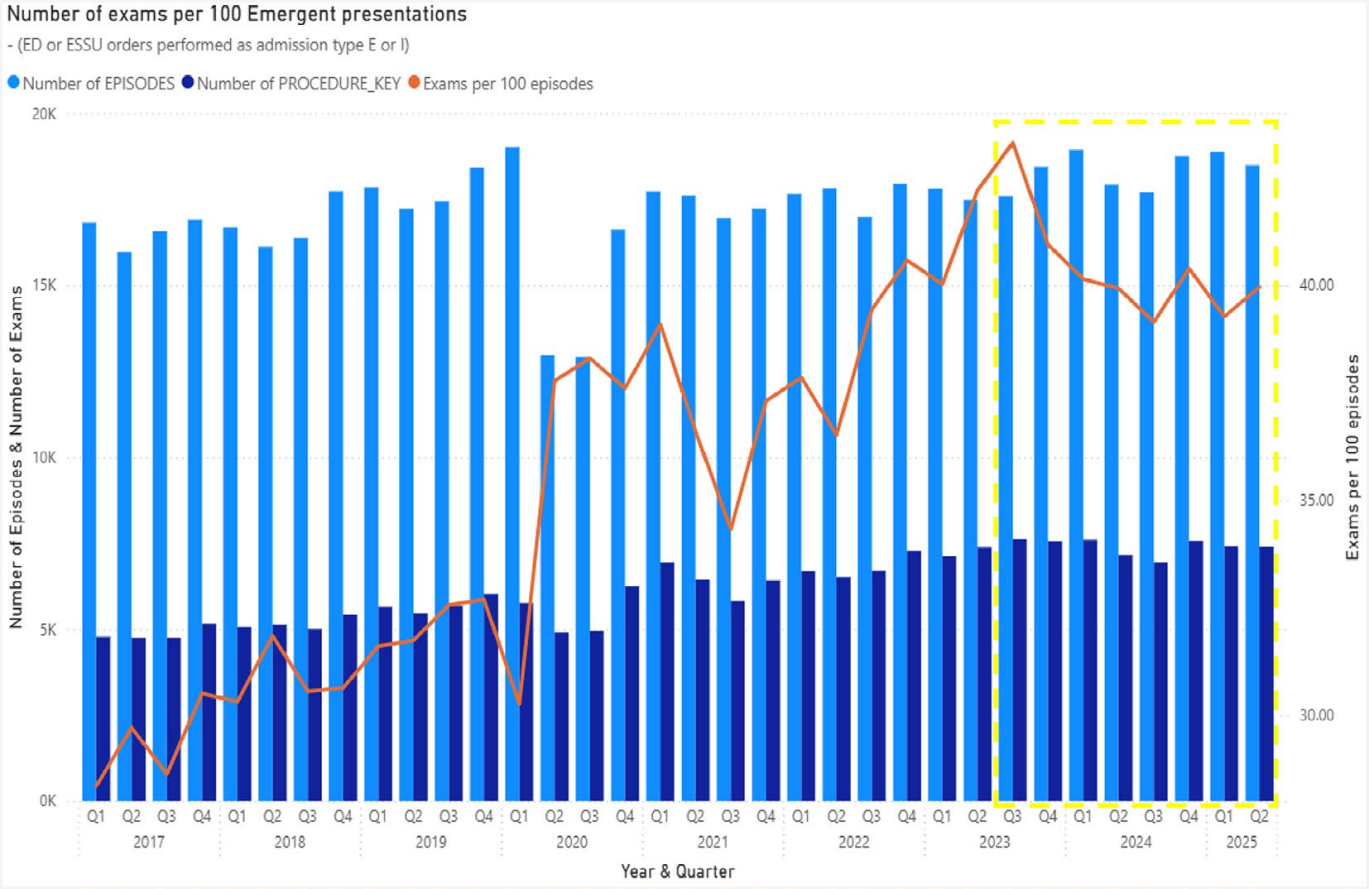
Quarterly CT utilisation rates per 100 emergency department (ED) presentations across the pre‐COVID baseline, pre‐ go‐live engagement phase and eCDS deployment phase. The period spanning the engagement and deployment phases (Q3 2023 to end June 2025) is highlighted in yellow.

As noted earlier, one of the key drivers for implementing eCDS was the rising trend in imaging utilisation. Prior to the pre‐go‐live engagement phase, CT examinations increased from 32 per 100 ED presentations (pre‐COVID) to 44 per 100 in 2023, underscoring the need for intervention. Interestingly, a decline in CT ordering was observed even before the decision support tool went live, coinciding with the initial education and awareness campaign (Figure 4). During the pre‐go‐live engagement phase, CT utilisation decreased from 44 to 40 examinations per 100 ED presentations, representing a 9.1% reduction. This suggests that changing clinician mindset, rather than relying solely on eCDS automation, may influence imaging behaviour.

Imaging utilisation trends remained stable following eCDS deployment. Although a further sustained reduction in CT utilisation was not observed following eCDS go‐live, maintenance of lower utilisation rates during the deployment phase may suggest persistence of behavioural and organisational changes established during the pre‐go‐live engagement phase.

## 4 Discussion

### 4.1 Cultural Change and Clinician Engagement

One of the most notable observations during the eCDS implementation was a cultural shift towards more judicious imaging use. Rather than simply introducing a digital tool, continuous engagement and education fostered deeper understanding and sustained adoption of evidence‐based imaging practises.

In the United States, mandates requiring eCDS use for Medicare reimbursement have shown some early success; however, meaningful engagement depends on clinicians understanding the rationale behind decision support systems rather than simply complying with mandates [26, 27]. Our experience suggests that whilst policy frameworks can promote initial uptake, lasting change requires cultural alignment and clinician buy‐in.

Ultimately, the strongest incentive for reducing low‐value imaging may be recognition of its broader impact, not only on patient safety and resource utilisation but also on environmental sustainability. In an era where climate change threatens planetary health, aligning clinical practise with sustainability principles provides a compelling reason for stakeholders to act responsibly [28].

These findings align with implementation science literature demonstrating that behavioural change strategies, including clinician engagement and feedback mechanisms, are critical determinants of successful adoption of clinical decision support systems [7, 10, 15].

### 4.2 Limitations and Future Directions

This study represents a single‐centre experience and reports observational trends rather than causal effects of the intervention. Changes in imaging utilisation may also have reflected concurrent education initiatives, organisational change processes and broader institutional influences in addition to eCDS deployment. Inferential statistical modelling was not performed, limiting attribution of observed effects specifically to the eCDS platform.

CT utilisation per ED presentation represents an indirect surrogate measure of eCDS impact and does not fully capture imaging appropriateness or decision quality across referral pathways. Although operational appropriateness analytics derived from the eCDS platform were available for ongoing monitoring, these metrics were not formally analysed as primary study outcomes. In addition, the present analysis did not evaluate detailed cancellation pathways or reasons for imaging order modification at the eCDS or downstream workflow level.

Future work should focus on broader organisational evaluation across Australian healthcare settings, including additional specialties, imaging modalities and appropriateness metrics derived from eCDS analytics. Incorporation of clinician feedback loops and adaptation of local guideline pathways may further improve usability, adoption and clinical impact.

Integration of AI‐enabled tools within EMR and eCDS platforms may further enhance decision support capabilities and workflow optimisation. Alignment with statewide and national quality initiatives, including the SQuDS programme, may support sustainability and scalability of future eCDS implementation strategies.

## 5 Conclusions

The experience at Alfred Hospital suggests that successful integration of eCDS into clinical practise requires more than technological deployment alone. Sustainable change appears to depend on broader organisational and cultural shifts in clinical decision‐making. Passive deployment is unlikely to achieve lasting impact, whereas active change management strategies—including clinician education, governance structures, real‐time analytics and feedback mechanisms—may facilitate clinician engagement and support adoption of decision support systems.

## Funding

The project was supported by departmental resources within Alfred Health.

## Ethics Statement

Ethics approval was obtained from the Alfred Health Ethics Committee. The study was approved under Ethics Committee Project No. 163/25.

## Conflicts of Interest

The authors declare no conflicts of interest.

## Data Availability

The data that support the findings of this study are available from the corresponding author upon reasonable request.
